# LDL Cholesterolemia as a Novel Risk Factor for Radiographic Progression of Rheumatoid Arthritis: A Single-Center Prospective Study

**DOI:** 10.1371/journal.pone.0068975

**Published:** 2013-07-29

**Authors:** Yune-Jung Park, Chul-Soo Cho, Paul Emery, Wan-Uk Kim

**Affiliations:** 1 Divsion of Rheumatology, Department of Internal Medicine, The Catholic University of Korea, School of Medicine, Seoul, Korea; 2 Division of Musculoskeletal Disease, Leeds Institute of Rheumatic and Musculoskeletal Medicine, University of Leeds, Chapel Allerton Hospital, Leeds, United Kingdom; 3 NIHR Leeds Musculoskeletal Biomedical Research Unit, Leeds Teaching Hospitals NHS Trust, Leeds, United Kingdom; University of Leicester, United Kingdom

## Abstract

Dyslipidemia has been implicated in various musculoskeletal diseases, including rheumatoid arthritis (RA). Evidence is emerging that there might be a pathogenic interaction among inflammation, dyslipidemia, and adipokines. We prospectively investigated the association of cumulative lipid levels with radiographic progression of RA. RA patients (n = 242) underwent plasma cholesterol assessment at four visits. Disease activity parameters and X-rays of the hands and feet were also serially monitored in these patients. The cumulative inflammatory burden and lipid levels were estimated by time-integrated values. Serum leptin and adiponectin concentrations were determined by ELISA. When patients were divided into three groups according to time-integrated lipid levels, as expected, patients with LDL cholesterol and/or triglyceride levels in the third tertile had persistently higher ESR and CRP levels. In parallel, a more rapid radiographic progression over two years was observed in patients with higher LDL cholesterol and/or triglyceride levels. In multivariate analysis, time-integrated LDL cholesterol was independently associated with radiographic progression. Particularly, the risk of radiographic progression was 5.6-fold in a subgroup with both LDL cholesterol and triglyceride levels in the third tertile. Moreover, LDL cholesterol synergistically increased the adjusted probability of radiographic progression in patients with high serum leptin levels but not in those without. These results demonstrate that LDL cholesterolemia is a novel serum marker that can be used to predict radiographic progression of RA, which seems to be related to circulatory leptin levels. We suggest that personalized and more aggressive anti-rheumatic therapy is required for dyslipidemic subgroups in RA patients.

## Introduction

Dyslipidemia has been associated with a variety of types of inflammatory arthritis such as gout and familial hypercholesterolemia with migratory polyarthritis [1]. In regard to arthritis, earlier studies have demonstrated that obese people are at an increased risk of developing rheumatoid arthritis (RA) and psoriatic arthritis [2]–[4]. Two recent studies [5], [6] also reported that lipid-lowering therapy may be beneficial in reducing disease activity and the number of swollen joints in RA. Moreover, oxidized low density lipoprotein (LDL) is increased in inflamed synovial fluid [7], and both intracellular and extracellular oxidized LDL are detected in the rheumatoid synovium [8]. Recently, we have shown that there is a common genetic predisposition for the synchronicity of RA and dyslipidemia [9], [10]. Together, these observations suggest that dyslipidemia is linked to the pathogenesis of RA.

In comparison with general population, patients with RA have higher prevalence of dyslipidemia [11]. Although not all studies are consistent, patients with active untreated RA demonstrate reduced high density lipoprotein (HDL), but increased LDL cholesterol and lipoprotein(a) levels [12]. In this regard, systemic inflammation can be a notable contributor to the lipid profile changes [13]. In contrast, evidence that lipids have a direct modulating effect on inflammation has emerged. For example, hypercholesterolemia induces inflammation by increasing circulating inflammatory cells [14], [15]. Other studies have demonstrated an association between oxidized LDL cholesterol and proinflammatory cytokines such as interleukin-6 (IL-6) and tumor necrosis factor-α (TNF-α) [16]. Adipose tissue also secretes various cytokines such as the proinflammatory TNF-α and IL-6 [17], which are key cytokines responsible for the perpetuation of RA. Most studies on the relationship between rheumatoid inflammation and dyslipidemia have been limited in that they were performed in animal models of chronic arthritis or were not analyzed in a prospective manner. Therefore, it is unclear whether dyslipidemia has pathogenic or phenotypic correlations with RA activity and severity in humans.

An accumulation of chronic synovitis over time results in progressive deformity in RA, which leads to irreversible disability [18]. Therefore, the discovery of prognostic biomarkers is important for the early identification of patients with a potentially aggressive disease course and the development of tailored aggressive therapy. Risk factors already established to predict a more rapid progression include the presence of anti-cyclic citrullinated peptide antibody (ACPA) and rheumatoid factor (RF), the shared epitope, and chronically elevated synovial and systemic inflammation [19]. However, with the exception of the inflammatory markers, these risk factors cannot be easily modified. Thus, it is important to find reversible and measurable biomarkers of radiographic progression. In this study, we postulate that the presence and/or severity of dyslipidemia may reflect the disease activity of RA, and thus lipid levels could be an additional biomarker for the progression of RA since they apparently correlate well and independently with the degree of rheumatoid inflammation [12], [20], which is one of the strongest prognostic factors of RA. To address this issue, we prospectively investigated the tripartite relationship among cholesterol levels and inflammatory markers/disease activity, which were regularly monitored over two years, and radiographic progression of RA.

## Materials and Methods

### Ethics Statement

The study protocol was approved by the Institutional Review Board of the Catholic Medical Center (VC12RISI0191). All patients gave written informed consent to the study protocol.

### Patients' Clinical Characteristics

The hospital-based study group was composed of 324 consecutive RA patients. All participants fulfilled the 1987 American College of Rheumatology criteria for the classification of RA [21]. Forty-eight RA patients failed to provide written informed consent, leaving 276 RA patients enrolled in this study. An additional 32 of those 276 patients had one or more missing values and were subsequently excluded. In total, 242 RA patients with complete data were analyzed for this study. The following subjects were excluded: those with severe cardiac, renal, or nutritional disorders that would affect lipid levels, current or chronic infection, pregnancy, excessive alcohol use (>5 times per week), and a history of malignancy. Information on demographic characteristics was collected, including age, gender, body mass index (BMI), presence of hypertension or diabetes mellitus, disease duration, positive RF detection, positive ACPA detection, disease activity, and disease severity. Disease activity was evaluated with a Disease Activity Score 28-joint assessment (DAS28) [22]. Disease severity was assessed by evaluating radiographic damage on X-rays of the hands and feet. Current medication use was carefully recorded both from the information provided by the patients and from medical records, including disease modifying anti-rheumatic drugs (DMARD), biologics, and the dose and type of glucocorticoids.

### Radiographic Assessment

Radiographs of the hands and feet were taken at baseline and annually thereafter. The radiographic severity was scored in chronologic order for erosions and joint space narrowing according to the Sharp/van der Heijde (SvdH) method [23] and was determined by two board-certified physicians who were blinded to each patient’s identity and clinical status. The inter-observer variability described by the interclass correlation coefficient was 0.93. Joint space narrowing and erosion scores were summed to give the total radiographic progression score. Erosion and narrowing progression scores were calculated by subtracting the initial score from the score after a two-year follow-up. Radiographic progression was defined as a progression score ≧4 [19].

### Measurement of Plasma Lipid Levels and Adipocytokine Levels

Fasting blood samples were collected at each study visit (total of four repeated measures per participants). Lipid parameters were measured according to standard procedures at the Department of Clinical Chemistry, St. Vincent’s Hospital, The Catholic University of Korea. Plasma total cholesterol and triglyceride levels were assayed using enzymatic CHOD-PAP methods (Roche Diagnostics, Meylan, France). HDL cholesterol levels were measured using a homogenous method based on the polyanion-polymer/detergent (Daiichi, Tokyo, Japan). LDL cholesterol was calculated by the Friedewald formula, with the assignment of missing values to subjects with a triglyceride level <400 mg/dl. We considered the patients to have dyslipidemia if their fasting plasma HDL cholesterol was <40 mg/dl in men and <50 mg/dl in women or if their LDL cholesterol was ≥130 mg/dl, their triglyceride was ≥200 mg/dl, or their total cholesterol was ≥200 mg/dl. Serum samples were obtained at baseline and stored at −70°C. The serum concentrations of adipocytokines (leptin [ng/ml] and adiponectin [µg/ml]) were measured by a commercial enzyme-linked immunosorbent assay (ELISA) kit (R&D Systems, Minneapolis, MN, USA), according to the manufacturer’s recommendations.

### Statistical Analyses

The distributions of all variables were examined. Cumulative inflammatory burden and cholesterol levels were expressed in time-integrated values, calculated from the area under the curve for each patient during the two-year follow-up [19]. Comparisons between the radiographic progression group and non-progression group were performed by the independent t-test and the Mann–Whitney test, and the χ2 test was used for means, medians, and proportions. Correlations were performed by Spearman correlation analysis. To evaluate effects on cholesterol levels in serially monitored inflammatory markers, repeated measures one-way analysis of variance (ANOVA) were used. Multivariable logistic regression was performed to analyze for significant and independent contributions to radiographic progression. We used radiographic progression events (any increase in SvdH score ≥4) as the outcome after determining the presence of radiographic progression at two years. Multivariate models were constructed that included all covariates with associations from univariate models with a *P*-value ≤0.20. Cumulative probability plots were used to display radiographic progression across patients with different baseline levels of adipokines. Receiver operating characteristic (ROC) analyses were performed to find the optimal cut-off levels for adiponectin and leptin to discriminate between high and low. High leptin and high adiponectin were defined when serum leptin levels were ≥16.888 ng/ml and serum adiponectin levels were ≥1.682 µg/ml. All reported *P*-values are two-tailed, with a *P*-value of 0.05 indicating statistical significance. The sample size was calculated to achieve a power of 80% via the following hypotheses: 1) a prevalence of at least 0.20 of radiographic progression in the highest tertile group of time-integrated LDL cholesterol; and 2) an odds ratio of 2.0 associated with the studied variable for the outcome (α = 0.05). Under these suppositions, the calculated total sample size was 221. The odds ratio was identified by logistic regression. Analyses were performed with the use of SAS software, version 9.2 (SAS Institute Inc., NC, USA).

## Results

### Baseline Characteristics

Plasma levels of cholesterol were measured in 242 patients with RA. The mean age was 53.8 years, and the median disease duration was 6 years (**Table S1**). One hundred eighty-nine (78.1%) patients were positive for ACPA, and the median SvdH score was 31 [IQR:13-78]. One hundred eighty-seven patients (77.3%) received methotrexate, 155 (64.0%) received hydroxychloroquine, 26 (10.7%) received anti-TNF-α therapy, and 187 (77.3%) patients were treated with a low dose of prednisolone (≤10 mg/day). Other characteristics of the RA patients are shown in **Table S1**. As reported previously [24]–[26], when we compared plasma lipid levels between RA patients and age- and gender-matched healthy controls, LDL cholesterol levels and triglyceride levels were increased, but HDL cholesterol levels were decreased in RA patients (**Figure S1**).

### Dyslipidemia and RA Disease Activity

**Figure 1:**
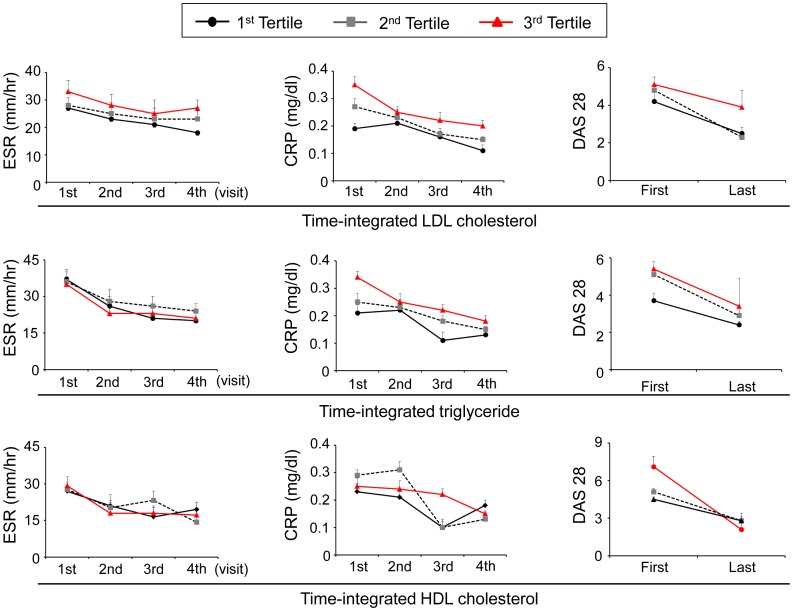
Changes in ESR, CRP level, and DAS28 during the follow-up period according to time-integrated lipid tertile. Patients with LDL cholesterol levels in the third tertile had persistently higher ESR levels (main effect of group: *P*<0.001, main effect of time: *P*<0.001, interaction effect: *P*<0.001), CRP levels (main effect of group: *P*<0.001, main effect of time: *P*<0.001, interaction effect: *P*<0.001), and DAS28 scores (main effect of group: *P* = 0.014, main effect of time: *P* = 0.016, interaction effect: *P*<0.001) than those with levels in the first tertile. Patients with triglycerides levels in the third tertile had higher CRP levels (main effect of group: *P* = 0.021, main effect of time: *P*<0.001, interaction effect: *P*<0.001) and DAS28 scores (main effect of group: *P* = 0.011, main effect of time: *P* = 0.016, interaction effect: *P*<0.001) than those with levels in the first tertile. No difference was found in ESR, CRP, and DAS28 according to HDL cholesterol levels. Values are expressed as mean±SD. *P*-values are calculated by ANOVA repeated measures. See the Table 1 for abbreviations.

Since lipid levels are influenced by disease activity [12], we studied whether lipid levels correlated with RA activity in our cohort. When patients were divided into three tertile groups according to time-integrated lipid levels, patients with LDL cholesterol levels in the third tertile had persistently higher erythrocyte sedimentation rate (ESR), C-reactive protein (CRP), and DAS28 levels than the first tertile group (**Figure 1**). The patients with triglyceride levels in the third tertile also had higher baseline CRP and DAS28 levels than the first tertile group; however, there was no significant difference across the HDL tertiles. Interestingly, although the three tertile groups of LDL cholesterol and triglyceride showed a decreasing tendency of disease activity with anti-rheumatic treatments, this decreasing trend was significantly attenuated in the highest tertile group (**Figure 1**). These results suggest that patients with higher LDL cholesterol and triglyceride levels are in a more active state of disease, showing a poor response to medical treatment.

### Dyslipidemia and Radiographic Progression

We studied further whether lipid tertile could reflect radiographic progression. The result showed that the radiographic score increased with increasing LDL cholesterol and triglyceride tertile. Moreover, rapid progression was observed in the highest LDL cholesterol group (**Table S2**). In univariate analyses for the determination of factors affecting radiographic progression and time-integrated LDL cholesterol, disease duration, the presence of ACPA and RF, time-integrated CRP, time-integrated ESR, and the use of methotrexate were found to be statistically significant (**Table 1** and **Table S3**, respectively). These variables were therefore included in the multivariate logistic models (**Table 2**). As shown in **Table 2**, time-integrated LDL cholesterol levels were independently associated with radiographic progression (highest tertile versus lowest tertile OR = 2.831, 95% CI: [1.561–4.246], *P* = 0.031); however, neither time-integrated triglyceride nor HDL levels were found to significantly increase the risk of radiographic progression of RA. Since TNF-α blocking agents and statin therapy can affect radiographic progression and LDL cholesterol, respectively [5], [6], [27], we further analyzed the potential effects of these agents. The association between radiographic progression and LDL cholesterol levels remained significant after adjustment for the use of TNF-α inhibitors (OR = 2.194 [1.532–3.141], *P* = 0.039) and the use of statin (OR = 3.124, *P* = 0.001). In addition, radiographic progression rate may be also influenced by the baseline radiographic severity. Thus, we further performed linear regression analysis of the absolute radiographic scores as a dependent variable after adjusting the baseline radiographic score. When the multivariate regression analysis was performed after adjusting for confounders, time-integrated LDL cholesterol remained an independent risk factor for radiographic severity at 24 weeks (**Table S4**).

**Table 1:** Association between patient characteristics and radiographic progression at two years.

Variables	RA patients with radiographicprogression (n = 61)	RA patients without radiographicprogression (n = 181)	*P*-value†
Age, years	54 (49–61)	52 (45–62)	0.113
Female, n (%)	50 (82.0)	138 (75.8)	0.271
Body mass index, kg/m^2^	22.1 (19.9–24.2)	23.1 (20.6–25.4)	0.074
Disease duration, years	8 (3–17)	6 (3–11)	0.044
Rheumatoid factor§, n (%)	48 (78.7)	117 (64.6)	0.042
ACPA§, n (%)	55 (90.2)	134 (73.6)	0.007
DAS28	4.4 (3.1–5.5)	3.9 (2.9–5.3)	0.137
Baseline ESR, mm/hour	31 (16–61)	22 (12–39)	0.052
Time-integrated ESR	1068 (558–2040)	720 (384–1272)	0.012
Baseline CRP, mg/dl	0.43 (0.09–1.57)	0.21 (0.08–0.82)	0.041
Time-integrated CRP	24.9 (5.1–90.5)	10.3 (2.4–33.4)	0.004
Methotrexate, n (%)	53 (86.9)	134 (74.0)	0.038
Anti-TNF α, n (%)	7 (11.5)	19 (10.5)	0.823
Time-integrated HDL cholesterol	1344 (1146–1488)	1272 (1080–1512)	0.306
Time-integrated triglyceride	2280 (1668–3336)	2112 (1560–2280)	0.040
Time-integrated LDL cholesterol	3098 (2557–3886)	2798 (2318–3182)	0.005

Data are presented as median (interquartile range) or number (%).

† *P*-values represent the comparison between the radiographic progression group and non-progression group.

§ = antibody positivity. ACPA = anti-cyclic citrullinated peptide antibody, DAS28 = disease activity score in 28 joints, ESR = erythrocyte sedimentation rate, CRP = C-reactive protein, TNF-α = tumor necrosis factor alpha, HDL = high density lipoprotein, and LDL = low density lipoprotein. The positive cut-off value for ACPA was ≧5 U/ml.

**Table 2:** Logistic regression analysis of radiographic progression at two years according to lipid levels.

Models	OR [95% CI]	*P-*value
Basic model+one kind of dyslipidemia		
Time-integrated low-density lipoprotein cholesterol, tertile		
Lowest	1	
2	1.604 [0.726–3.306]	0.173
3	2.831 [1.561–4.246]	0.031
Time-integrated triglyceride, tertile		
Lowest	1	
2	1.324 [0.635–2.561]	0.212
3	1.814 [0.998–3.791]	0.059
Time-integrated high-density lipoprotein cholesterol, tertile		
Lowest	1	
2	0.760 [0.049–1.352]	0.732
3	1.124 [0.898–2.818]	0.096

OR = odds ratio, CI = confidence interval. Basic model is comprised of age, body mass index, disease duration, presence of anti-cyclic citrullinated peptide antibody and rheumatoid factor, time-integrated erythrocyte sedimentation rate levels, time-integrated C-reactive protein levels, and methotrexate use.

**Figure 2:**
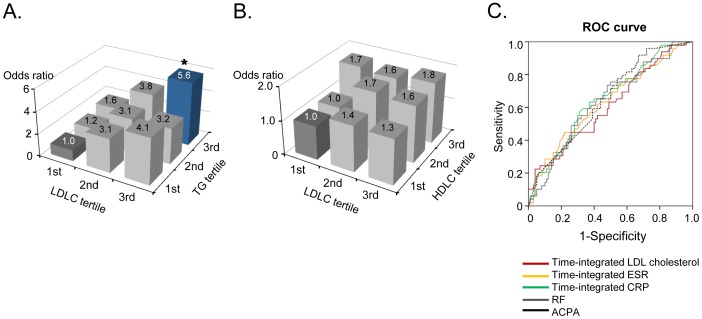
Comparison of the effects of LDL cholesterol levels, other lipid levels, and conventional prognostic factors on radiographic progression of rheumatoid arthritis. (**A**) Adjusted odds ratio of radiographic progression with combination of time-integrated LDL cholesterol and triglyceride levels. (**B**) Adjusted odds ratio of radiographic progression with combination of time-integrated LDL cholesterol and HDL cholesterol levels. The first tertile of lipid levels (dark gray column) was the reference group. All were adjusted for the following variables: age, body mass index, disease duration, presence of ACPA and RF, time-integrated ESR levels, time-integrated CRP levels, and methotrexate use. *P*-values are obtained from comparison with the reference group. *P<0.05 for the blue column. (**C**) Receiver operating characteristic curves of time-integrated LDL cholesterol, time-integrated ESR levels, time-integrated CRP levels, RF, and ACPA for prediction of radiographic progression. Area under the curve was 0.609 for time-integrated LDL cholesterol, 0.631 for ESR, 0.648 for CRP, 0.634 for RF, and 0.648 for ACPA. See the Table 1 for abbreviations.

To analyze the effect of other lipid levels on disease acceleration linked to LDL cholesterolemia, we divided the patients into nine groups based on three tertiles of LDL cholesterol, triglyceride, and HDL cholesterol (**Figure 2A and 2B**). Patients with both LDL cholesterol and triglyceride levels in the lowest tertile (dark gray column in the first row) were considered the reference subgroup. As a result, the adjusted odds ratios of radiographic progression additively rose as triglyceride tertile increased (**Figure 2A**). Particularly, in a subgroup with both LDL cholesterol and triglyceride levels in the third tertile, the adjusted odds ratio was 5.60 (95% CI: [1.25–7.14], *P* = 0.013), as compared to the reference subgroup (**Figure 2A**). In contrast, when the LDL cholesterol tertile was similarly analyzed in association with the HDL cholesterol tertile, such an increase in radiographic progression was not noted (**Figure 2B**). In fact, the adjusted odds ratios affected by HDL cholesterol tertile were 1.0 to 1.7 in all nine subgroups, which were much lower than the third tertile of LDL cholesterol only (OR = 2.831), suggesting that HDL cholesterolemia is rather protective for radiographic progression linked to LDL cholesterolemia. Together, these data indicate that LDL cholesterolemia interacts with triglyceridemia and HDL cholesterolemia for RA progression.

We next wanted to compare the influence of LDL cholesterolemia with that of conventional risk factors for RA progression, including time-integrated ESR, time-integrated CRP, the presence of rheumatoid factor, and the presence of ACPA. To address this issue, we evaluated the sensitivity and specificity of the time-integrated LDL cholesterol levels in comparison with conventional factors. When the ROC curve for each variable was analyzed, the area under the curve (AUC) of time-integrated LDL cholesterol was 0.609 [95%CI: 0.569–0.720], which was comparable to that of the time-integrated CRP (0.648, [0.536–0.684]), time-integrated ESR (0.631, [0.528–0.711]), RF (0.634, [0.547–0.688]), and ACPA (0.648, [0.537–0.683]) (**Figure 2C**). No difference in AUC was found between time-integrated LDL cholesterol and time-integrated CRP (*P* = 0.533). In addition, on the basis of the null distribution of AUC (100,000 random permutation of data), one-tailed *P* values for all variables were *P*<0.005. These results suggest that cumulative LDL cholesterolemia helps clinicians to predict disease progression as efficiently as conventional prognostic factors of RA.

### LDL Cholesterolemia, Adipocytokines, and Disease Progression

Evidence is emerging that adipocytokines with pro-inflammatory activity, mainly produced from adipose tissues, are increased in RA patients [17], [28], [29], and their levels correlate with disease activity and radiographic progression [18], [19], [30]–[34]. Our findings that LDL cholesterol showed an independent association with radiographic progression prompted us to investigate whether adipocytokines, including leptin and adiponectin, are involved in this association. The results showed that both adiponectin (log transformed value:γ = 0.234, *P* = 0.001) and leptin (log transformed value: γ = 0.211, *P* = 0.002) levels showed positive correlations with radiographic severity (**Figure S2A and S2B**). Moreover, serum leptin concentrations also correlated well with LDL cholesterol levels (log transformed value: γ = 0.294, *P*<0.001, **Figure S2C**).

**Figure 3:**
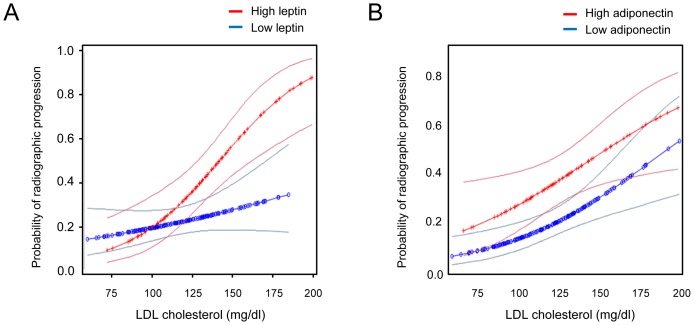
Effect of serum leptin levels on radiographic progression linked to LDL cholesterolemia. Adjusted cumulative probability plots for radiographic progression according to LDL cholesterol levels in patients with RA were stratified again by leptin levels (**A**) and adiponectin levels (**B**). In each panel, the upper cut-off value is represented by the red line (≧16.888 ng/ml for leptin and ≧1.682 µg/ml for adiponectin), and the lower cut-off value is the blue line (<16.888 ng/ml for leptin and <1.682 µg/ml for adiponectin). The upper and lower 95% confidence limits are depicted by the grey lines surrounding the average probability plot.

We further investigated whether serum adipokines affect the radiographic progression of RA linked to LDL cholesterolemia. To this end, we stratified the patients depending on serum leptin or adiponectin concentrations. As seen in **Figure 3A** **,** patients with high serum leptin (≧16.888 ng/ml) showed a higher adjusted probability of radiographic progression than those with low leptin (< 16.888 ng/ml). The increase in radiographic progression by high leptin was synergistic with LDL cholesterolemia (*P*<0.001). Interestingly, LDL cholesterolemia significantly increased radiographic progression in patients with high leptin levels (OR = 1.035, 95%CI: [1.016–1.033], *P*<0.001) but not in those with low leptin levels (OR = 1.010 [0.996–1.023], *P* = 0.167), demonstrating a dichotomy of leptin effect on radiographic progression under the conditions of LDL cholesterolemia (**Figure 3A**). In contrast to leptin, there was no difference in the effect of high (≧1.682 ng/ml) versus low adiponectin (<1.682 ng/ml) on radiographic progression (OR = 1.020 [1.003–1.037] and *P* = 0.018 for the high adiponectin subgroup; OR = 1.022 [1.009–1.036] and *P* = 0.001 for the low adiponectin subgroup) (**Figure 3B**). Moreover, the effect of adiponectin in both subgroups was additive but not synergistic (*P* = NS).

## Discussion

Inflammatory cytokines play a pathogenic role in abnormalities of lipid metabolism in a variety of disorders, including diabetes, obesity, metabolic syndrome, and atherosclerosis [35]. In regard to RA, reduced HDL cholesterol and elevated lipoprotein(a) correlated with elevated serum CRP levels and inflammatory activity [24]. Inflammatory activation may also drive higher LDL cholesterol levels in RA [24], [25]. Moreover, effective control of RA can thus reverse adverse lipid profiles [20]. Previous studies have demonstrated the relationship between disease activity and cholesterol levels, showing that initial total cholesterol levels could predict radiological progression in RA [36], [37]. However, which lipid component among LDL cholesterol, triglyceride, and HDL cholesterol is associated with radiographic progression is inconclusive. In addition, there is a paucity of prospective reports demonstrating a possible link between cumulative dyslipidemia and inflammatory activity and disease severity of RA.

In our longitudinal study, RA patients with persistently higher LDL cholesterol levels were in a persistently elevated inflammatory state, as determined by time-integrated ESR, time-integrated CRP, and DAS28, which is consistent with previous reports [12], [14]–[16], [20], [24]–[26], [38] that the inflammatory milieu adversely affects lipid profiles in RA. One of the most widely used lipid biomarkers is LDL cholesterol, which has been used in risk calculations for heart disease for over 50 years. Triglycerides are also used clinically for risk assessment of heart disease and diabetes. In RA patients, lipid and its derivatives, including prostaglandins (lipid compounds), adipokines, and fat-derived cytokines, function as biomarkers of disease activity and bone destruction [18], [19], [31]–[34], [38]. Since lipid levels can reflect the degree of chronic inflammation, it can be hypothesized that cumulative dyslipidemia reflects the radiographic severity of RA; however, no trial has been done to identify the direct association of dyslipidemia itself with RA progression. In the present study, we found that RA patients with chronically elevated LDL cholesterol levels had a 2.8-fold greater odds ratio of radiographic progression than those without, even after adjusting for potential confounders. Particularly, the risk of radiographic progression was 5.6-fold in a subgroup with both LDL cholesterol and triglyceride levels in the third tertile. The influence of LDL cholesterolemia on radiographic progression was comparable to that of conventional prognostic factors of RA, including ESR, CRP, the presence of RF, and the presence of ACPA. Our results suggest that cumulative dyslipidemia, especially LDL cholesterolemia, is a novel serum marker to predict radiographic progression of RA as well as cardiovascular complications.

Recently, we have demonstrated that there is a common genetic predisposition for synchronicity of RA and dyslipidemia [9], [10]. Of note, genes modulating LDL cholesterol levels were found to influence the inflammatory process, promoting radiographic severity of RA [9], [10]. In the present study, LDL cholesterolemia remained significant as a prognostic factor even after adjusting for inflammatory burden (e.g. time-integrated ESR/CRP levels), indicating that dyslipidemia itself contributed to radiographic progression in our patients. It is unclear how dyslipidemia is independently linked to RA progression. One possible explanation is that lipid itself might directly modulate chronic inflammation. Previous studies have shown that hyperlipidemic patients have more inflammation than non-dyslipidemic patients [39]. Additionally, obesity reduces the response rate to anti-TNF α therapy in RA [40] but increases the risk of developing RA [4]. Moreover, dyslipidemia can be present years before arthritis [5], [6]. In experimental models of arthritis, lipid residues are modified to crystalline cholesterol, and such crystals may serve as a chronic irritant in the joints [41]. Oxidized LDL is also found in inflamed synovial fluid [7], [42], initiating the vicious cycle of inflammation. Collectively, our data, together with previous reports [4], [7], [9], [10], [39], [40], [43], support the potential direct role of abnormal lipid profiles in RA progression, independent of inflammation.

Adipokines have been studied in relation to the pathogenesis of RA. They exert potent immune-modulatory actions on tissues and cells, including cartilage, synovium, bone, and various immune cells [17], [24], [28]. In RA, adipokine concentrations are increased and correlated with disease activity and joint destruction [18], [19], [29], [31]–[34]. In particular, leptin levels correlate well with synovial WBC counts [42], disease activity [29], and IL-17 [29]. In mice with antigen-induced arthritis, impaired leptin signaling via leptin or leptin-receptor deletion attenuates arthritis severity [44], demonstrating that leptin plays a direct role in RA progression. Furthermore, serum leptin concentrations closely correlate with plasma LDL cholesterol and triglyceride levels [45]. Consistent with previous reports [45], we found significant correlations between LDL cholesterol levels and serum leptin concentrations. Moreover, in conjunction with LDL cholesterolemia, high leptin and adiponectin levels increased the risk of radiographic progression synergistically and additively, respectively, suggesting that adipokines interact with LDL cholesterolemia for RA progression.

In summary, in a single-center prospective study, we found first that cumulative LDL cholesterolemia and/or triglyceridemia were associated with persistently high ESR and CRP levels. In parallel, a more rapid radiographic progression over two years was observed in patients with higher LDL cholesterol and/or triglyceride levels. In multivariate analysis, time-integrated LDL cholesterol was an independent risk factor for RA progression. The influence of LDL cholesterolemia on radiographic progression was comparable to that of conventional prognostic factors of RA such as CRP and seems to be related to serum adipokine concentrations. This study provides important evidence in humans that there may be a tripartite relationship among dyslipidemia, inflammatory activity, and disease progression of RA. Additionally, our data demonstrate that dyslipidemia, specifically cumulative LDL cholesterolemia, is a novel serum marker to predict radiographic progression of RA. We propose that more aggressive anti-rheumatic therapy is required for dyslipidemic subgroups in RA patients, not only to reduce cardiovascular complications but also to prevent rapid joint destruction and disability.

## Supporting Information

Figure S1 **Lipid profiles in RA patients versus control subjects.** Comparison of plasma LDL cholesterol, triglyceride, and HDL cholesterol levels between RA patients (n = 242) and age/sex-matched healthy controls (n = 242). The boundary of the box closest to zero indicates the 25th percentile, a line within the box marks the median, and the boundary of the box farthest from zero indicates the 75th percentile. Error bars above and below the boxes indicate the 90th and 10th percentiles, respectively. *P*-value was obtained by Wilcoxon rank sum test. **P<0.001.
(TIF)

Figure S2 **Relationship of serum adipokine concentrations with radiographic severity and LDL cholesterol levels.** (**A and B**) Correlation of serum leptin and adiponectin concentrations with radiographic severity. (**C and D**) Correlation of serum leptin concentrations with plasma LDL cholesterol levels.
(TIF)

Table S1 **Baseline characteristics of patients with RA.**
(DOCX)

Table S2 **Association between time-integrated lipid levels and radiographic severity and progression of rheumatoid arthritis.**
(DOCX)

Table S3 **Univariate analysis of clinical variables according to time-integrated LDL cholesterol levels.**
(DOCX)

Table S4 **Association between patient characteristics and radiographic severity at two years.**
(DOCX)

## References

[1] Struthers GR, Scott DL, Bacon PA, Walton KW (1983) Musculoskeletal disorders in patients with hyperlipidaemia. Ann Rheum Dis 42: 519–523. 6625701 10.1136/ard.42.5.519 PMC1001287

[2] Li W, Han J, Qureshi AA (2012) Obesity and risk of incident psoriatic arthritis in US women. Ann Rheum Dis 71: 1267–1272. 22562978 10.1136/annrheumdis-2011-201273 PMC4183754

[3] Love TJ, Zhu Y, Zhang Y, Wall-Burns L, Ogdie A, et al (2012) Obesity and the risk of psoriatic arthritis: a population-based study. Ann Rheum Dis 71: 1273–1277. 22586165 10.1136/annrheumdis-2012-201299 PMC3645859

[4] Symmons DP, Bankhead CR, Harrison BJ, Brennan P, Barrett EM, et al (1997) Blood transfusion, smoking, and obesity as risk factors for the development of rheumatoid arthritis: results from a primary care-based incident case-control study in Norfolk, England. Arthritis Rheum 40: 1955–1961. 9365083 10.1002/art.1780401106

[5] Jick SS, Choi H, Li L, McInnes IB, Sattar N (2009) Hyperlipidaemia, statin use and the risk of developing rheumatoid arthritis. Ann Rheum Dis 68: 546–551. 18662929 10.1136/ard.2008.091967

[6] McCarey DW, McInnes IB, Madhok R, Hampson R, Scherbakov O, et al (2004) Trial of Atorvastatin in Rheumatoid Arthritis (TARA): double-blind, randomised placebo-controlled trial. Lancet 363: 2015–2021. 15207950 10.1016/S0140-6736(04)16449-0

[7] James MJ, van Reyk D, Rye KA, Dean RT, Cleland LG, et al (1998) Low density lipoprotein of synovial fluid in inflammatory joint disease is mildly oxidized. Lipids 33: 1115–1121. 9870907 10.1007/s11745-998-0313-8

[8] Winyard PG, Tatzber F, Esterbauer H, Kus ML, Blake DR, et al (1993) Presence of foam cells containing oxidised low density lipoprotein in the synovial membrane from patients with rheumatoid arthritis. Ann Rheum Dis 52: 677–680. 8239763 10.1136/ard.52.9.677 PMC1005146

[9] Park YJ, Yoo SA, Choi S, Yoo HS, Yoon HS, et al. (2013) Association of Polymorphisms Modulating Low-density Lipoprotein Cholesterol with Susceptibility, Severity, and Progression of Rheumatoid Arthritis. J Rheumatol [*in press*]. 10.3899/jrheum.120954 23588940

[10] Park YJ, Yoo SA, Lee JH, Chung YJ, Cho CS, et al (2012) The APOM polymorphism as a novel risk factor for dyslipidaemia in rheumatoid arthritis: a possible shared link between disease susceptibility and dyslipidaemia. Clin Exp Rheumatol 31: 180–188. 23190940

[11] Yoo WH (2004) Dyslipoproteinemia in patients with active rheumatoid arthritis: effects of disease activity, sex, and menopausal status on lipid profiles. J Rheumatol 31: 1746–1753. 15338494

[12] Choy E, Sattar N (2009) Interpreting lipid levels in the context of high-grade inflammatory states with a focus on rheumatoid arthritis: a challenge to conventional cardiovascular risk actions. Ann Rheum Dis 68: 460–469. 19286905 10.1136/ard.2008.101964

[13] Myasoedova E, Crowson CS, Kremers HM, Roger VL, Fitz-Gibbon PD, et al (2011) Lipid paradox in rheumatoid arthritis: the impact of serum lipid measures and systemic inflammation on the risk of cardiovascular disease. Ann Rheum Dis 70: 482–487. 21216812 10.1136/ard.2010.135871 PMC3058921

[14] Drechsler M, Megens RT, van Zandvoort M, Weber C, Soehnlein O (2010) Hyperlipidemia-triggered neutrophilia promotes early atherosclerosis. Circulation 122: 1837–1845. 20956207 10.1161/CIRCULATIONAHA.110.961714

[15] Swirski FK, Libby P, Aikawa E, Alcaide P, Luscinskas FW, et al (2007) Ly-6Chi monocytes dominate hypercholesterolemia-associated monocytosis and give rise to macrophages in atheromata. J Clin Invest 117: 195–205. 17200719 10.1172/JCI29950 PMC1716211

[16] Tam LS, Tomlinson B, Chu TT, Li TK, Li EK (2007) Impact of TNF inhibition on insulin resistance and lipids levels in patients with rheumatoid arthritis. Clin Rheumatol 26: 1495–1498. 17237906 10.1007/s10067-007-0539-8

[17] Tilg H, Moschen AR (2006) Adipocytokines: mediators linking adipose tissue, inflammation and immunity. Nat Rev Immunol 6: 772–783. 16998510 10.1038/nri1937

[18] Giles JT, Allison M, Bingham CO, 3rd, Scott WM, Jr., Bathon JM (2009) Adiponectin is a mediator of the inverse association of adiposity with radiographic damage in rheumatoid arthritis. Arthritis Rheum 61: 1248–1256. 19714593 10.1002/art.24789 PMC2759038

[19] Giles JT, van der Heijde DM, Bathon JM (2011) Association of circulating adiponectin levels with progression of radiographic joint destruction in rheumatoid arthritis. Ann Rheum Dis 70: 1562–1568. 21571734 10.1136/ard.2011.150813 PMC3543946

[20] Park YB, Choi HK, Kim MY, Lee WK, Song J, et al (2002) Effects of antirheumatic therapy on serum lipid levels in patients with rheumatoid arthritis: a prospective study. Am J Med 113: 188–193. 12208376 10.1016/s0002-9343(02)01186-5

[21] Arnett FC, Edworthy SM, Bloch DA, McShane DJ, Fries JF, et al (1988) The American Rheumatism Association 1987 revised criteria for the classification of rheumatoid arthritis. Arthritis Rheum 31: 315–324. 3358796 10.1002/art.1780310302

[22] Prevoo ML, van 't Hof MA, Kuper HH, van Leeuwen MA, van de Putte LB, et al (1995) Modified disease activity scores that include twenty-eight-joint counts. Development and validation in a prospective longitudinal study of patients with rheumatoid arthritis. Arthritis Rheum 38: 44–48. 7818570 10.1002/art.1780380107

[23] van der Heijde D (2000) How to read radiographs according to the Sharp/van der Heijde method. J Rheumatol 27: 261–263. 10648051

[24] Dursunoglu D, Evrengul H, Polat B, Tanriverdi H, Cobankara V, et al (2005) Lp(a) lipoprotein and lipids in patients with rheumatoid arthritis: serum levels and relationship to inflammation. Rheumatol Int 25: 241–245. 15290086 10.1007/s00296-004-0438-0

[25] Hurt-Camejo E, Paredes S, Masana L, Camejo G, Sartipy P, et al (2001) Elevated levels of small, low-density lipoprotein with high affinity for arterial matrix components in patients with rheumatoid arthritis: possible contribution of phospholipase A2 to this atherogenic profile. Arthritis Rheum 44: 2761–2767. 11762936 10.1002/1529-0131(200112)44:12<2761::aid-art463>3.0.co;2-5

[26] Popa C, van den Hoogen FH, Radstake TR, Netea MG, Eijsbouts AE, et al (2007) Modulation of lipoprotein plasma concentrations during long-term anti-TNF therapy in patients with active rheumatoid arthritis. Ann Rheum Dis 66: 1503–1507. 17472994 10.1136/ard.2006.066191 PMC2111626

[27] Feldmann M, Maini RN (2001) Anti-TNF alpha therapy of rheumatoid arthritis: what have we learned? Annu Rev Immunol 19: 163–196. 11244034 10.1146/annurev.immunol.19.1.163

[28] Gomez R, Conde J, Scotece M, Gomez-Reino JJ, Lago F, et al (2011) What's new in our understanding of the role of adipokines in rheumatic diseases? Nat Rev Rheumatol 7: 528–536. 21808287 10.1038/nrrheum.2011.107

[29] Xibille-Friedmann D, Bustos-Bahena C, Hernandez-Gongora S, Burgos-Vargas R, Montiel-Hernandez JL (2010) Two-year follow-up of plasma leptin and other cytokines in patients with rheumatoid arthritis. Ann Rheum Dis 69: 930–931. 20413571 10.1136/ard.2009.111732

[30] Bokarewa M, Bokarew D, Hultgren O, Tarkowski A (2003) Leptin consumption in the inflamed joints of patients with rheumatoid arthritis. Ann Rheum Dis 62: 952–956. 12972473 10.1136/ard.62.10.952 PMC1754314

[31] Drossaers-Bakker KW, Zwinderman AH, Vliet Vlieland TP, Van Zeben D, Vos K, et al (2002) Long-term outcome in rheumatoid arthritis: a simple algorithm of baseline parameters can predict radiographic damage, disability, and disease course at 12-year followup. Arthritis Rheum 47: 383–390. 12209484 10.1002/art.10513

[32] Ebina K, Fukuhara A, Ando W, Hirao M, Koga T, et al (2009) Serum adiponectin concentrations correlate with severity of rheumatoid arthritis evaluated by extent of joint destruction. Clin Rheumatol 28: 445–451. 19085030 10.1007/s10067-008-1074-y

[33] Klein-Wieringa IR, van der Linden MP, Knevel R, Kwekkeboom JC, van Beelen E, et al (2011) Baseline serum adipokine levels predict radiographic progression in early rheumatoid arthritis. Arthritis Rheum 63: 2567–2574. 21567382 10.1002/art.30449

[34] Rho YH, Solus J, Sokka T, Oeser A, Chung CP, et al (2009) Adipocytokines are associated with radiographic joint damage in rheumatoid arthritis. Arthritis Rheum 60: 1906–1914. 19565493 10.1002/art.24626 PMC2894567

[35] Khovidhunkit W, Kim MS, Memon RA, Shigenaga JK, Moser AH, et al (2004) Effects of infection and inflammation on lipid and lipoprotein metabolism: mechanisms and consequences to the host. J Lipid Res 45: 1169–1196. 15102878 10.1194/jlr.R300019-JLR200

[36] Dawes PT, Fowler PD, Jackson R, Collins M, Shadforth MF, et al (1986) Prediction of progressive joint damage in patients with rheumatoid arthritis receiving gold or D-penicillamine therapy. Ann Rheum Dis 45: 945–949. 3539037 10.1136/ard.45.11.945 PMC1002025

[37] Wilding P, Kendall MJ, Holder R, Grimes JA, Farr M (1975) The influence of drugs and disease activity on biochemical and haematological data in rheumatoid arthritis. Clin Chim Acta 64: 185–194. 1183034 10.1016/0009-8981(75)90200-4

[38] Robinson DR, McGuire MB, Levine L (1975) Prostaglandins in the rheumatic diseases. Ann N Y Acad Sci 256: 318–329. 169722 10.1111/j.1749-6632.1975.tb36058.x

[39] Klaasen R, Wijbrandts CA, Gerlag DM, Tak PP (2011) Body mass index and clinical response to infliximab in rheumatoid arthritis. Arthritis Rheum 63: 359–364. 21279992 10.1002/art.30136

[40] Gremese E, Carletto A, Padovan M, Atzeni F, Raffeiner B, et al.. (2012) Obesity reduces the response rate to anti TNFalpha in rheumatoid arthritis. an approach to a personalized medicine. Arthritis Care Res (Hoboken). 10.1002/acr.21768 22730143

[41] Valente AJ, Walton KW (1980) Studies on increased vascular permeability in the pathogenesis of lesions of connective tissue diseases: I. Experimental hyperlipidaemia and immune arthropathy. Ann Rheum Dis 39: 490–499. 7436581 10.1136/ard.39.5.490 PMC1000592

[42] Seven A, Guzel S, Aslan M, Hamuryudan V (2009) Serum and synovial fluid leptin levels and markers of inflammation in rheumatoid arthritis. Rheumatol Int 29: 743–747. 19009296 10.1007/s00296-008-0764-8

[43] van Halm VP, Nielen MM, Nurmohamed MT, van Schaardenburg D, Reesink HW, et al (2007) Lipids and inflammation: serial measurements of the lipid profile of blood donors who later developed rheumatoid arthritis. Ann Rheum Dis 66: 184–188. 16760255 10.1136/ard.2006.051672 PMC1798498

[44] Busso N, So A, Chobaz-Peclat V, Morard C, Martinez-Soria E, et al (2002) Leptin signaling deficiency impairs humoral and cellular immune responses and attenuates experimental arthritis. J Immunol 168: 875–882. 11777985 10.4049/jimmunol.168.2.875

[45] Hamnvik OP, Liu X, Petrou M, Gong H, Chamberland JP, et al (2011) Soluble leptin receptor and leptin are associated with baseline adiposity and metabolic risk factors, and predict adiposity, metabolic syndrome, and glucose levels at 2-year follow-up: the Cyprus Metabolism Prospective Cohort Study. Metabolism 60: 987–993. 21056886 10.1016/j.metabol.2010.09.009

