# Supplementary figures and images for: LDL Cholesterolemia as a Novel Risk Factor for Radiographic Progression of Rheumatoid Arthritis: A Single-Center Prospective Study
Source: PLoS One. 2013 Jul 29;8(7):e68975. doi: 10.1371/journal.pone.0068975 (PMC3726747; doi:10.1371/journal.pone.0068975)

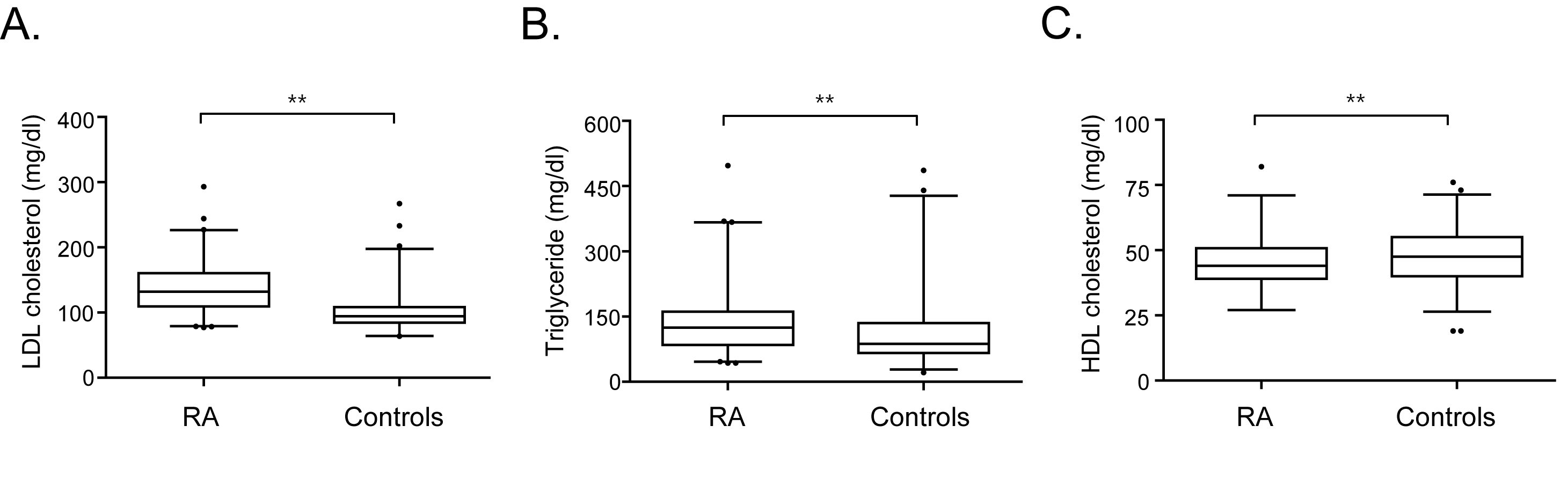

Supplement: Figure S1 — Lipid profiles in RA patients versus control subjects. Comparison of plasma LDL cholesterol, triglyceride, and HDL cholesterol levels between RA patients (n = 242) and age/sex-matched healthy controls (n = 242). The boundary of the box closest to zero indicates the 25th percentile, a line within the box marks the median, and the boundary of the box farthest from zero indicates the 75th percentile. Error bars above and below the boxes indicate the 90th and 10th percentiles, respectively. P-value was obtained by Wilcoxon rank sum test. **P<0.001. (TIF) [file pone.0068975.s001.tif]

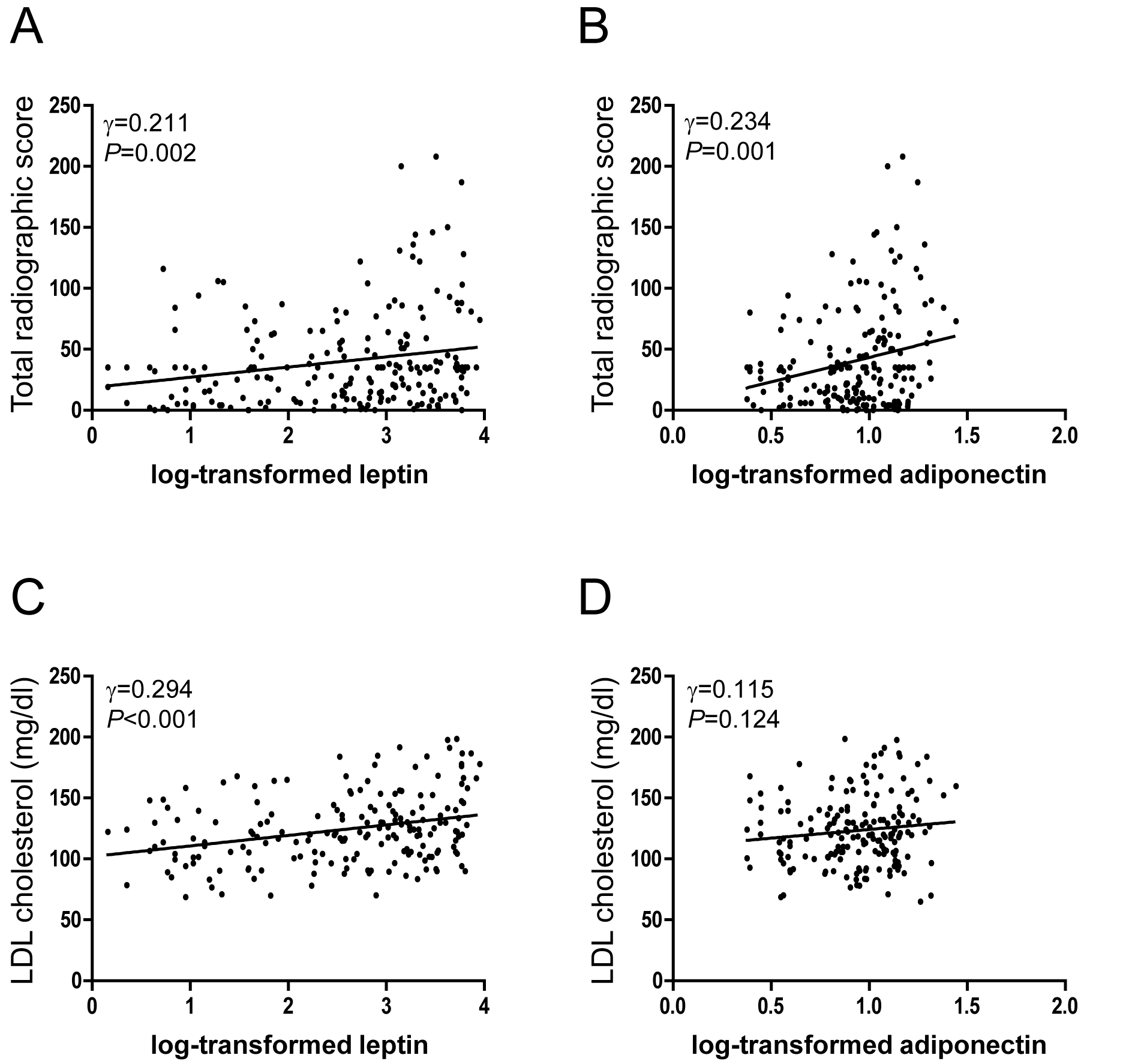

Supplement: Figure S2 — Relationship of serum adipokine concentrations with radiographic severity and LDL cholesterol levels. (A and B) Correlation of serum leptin and adiponectin concentrations with radiographic severity. (C and D) Correlation of serum leptin concentrations with plasma LDL cholesterol levels. (TIF) [file pone.0068975.s002.tif]
