# Supplementary material for: LDL Cholesterolemia as a Novel Risk Factor for Radiographic Progression of Rheumatoid Arthritis: A Single-Center Prospective Study
Source: PLoS One. 2013 Jul 29;8(7):e68975. doi: 10.1371/journal.pone.0068975 (PMC3726747; doi:10.1371/journal.pone.0068975)
Supplement: Table S1 — Baseline characteristics of patients with RA. (DOCX) [file pone.0068975.s003.docx]

**Table S1.** Baseline characteristics of patients with RA

| **Variables** | **Total patients**  (n=242) |
| --- | --- |
| Age, years | 53.8 ± 12.1 |
| Female, n (%) | 188 (77.7) |
| Body mass index, kg/m^2^ | 22.8 ± 3.3 |
| Total cholesterol, mg/dl | 216 ± 25.2 |
| Triglyceride, mg/dl | 134.2 ± 36.7 |
| High density lipoprotein cholesterol, mg/dl | 45.1 ± 5.3 |
| Low density lipoprotein cholesterol, mg/dl | 136.7 ± 18.9 |
| Disease duration, years | 6 (3-12) |
| Rheumatoid factor^†^, n (%) | 165 (68.2) |
| Anti-cyclic citrullinated peptide antibody^†^, n (%) | 189 (78.1) |
| Baseline erythrocyte sedimentation rate, mm/hour | 25 (13-44) |
| Baseline C-reactive protein, mg/dl | 0.24 (0.08-1.05) |
| Disease activity score in 28 joints | 4.1 (2.9-5.4) |
| Baseline total SvdH score | 31 (9-49) |
| Glucocorticoid, n (%) | 187 (77.3) |
| Hydroxychlroquine, n (%) | 155 (64.0) |
| Methotrexate, n (%) | 187 (77.3) |
| Anti-TNFα, n (%) | 26 (10.7) |
| Statin, n (%) | 29 (11.9) |

Data are presented as mean±SD, median (interquartile range), or number (%). The positive cut-off value for anti-cyclic citrullinated peptide antibody was ≧5 U/ml. SvdH=Sharp/van der Heijde and TNFα=tumor necrosis factor α. †=antibody positivity.
