# Supplementary material for: LDL Cholesterolemia as a Novel Risk Factor for Radiographic Progression of Rheumatoid Arthritis: A Single-Center Prospective Study
Source: PLoS One. 2013 Jul 29;8(7):e68975. doi: 10.1371/journal.pone.0068975 (PMC3726747; doi:10.1371/journal.pone.0068975)
Supplement: Table S2 — Association between time-integrated lipid levels and radiographic severity and progression of rheumatoid arthritis. (DOCX) [file pone.0068975.s004.docx]

**Table S2**. Association between time-integrated lipid levels and radiographic severity and progression of rheumatoid arthritis.

| Radiographic score | Time-integrated low-density lipoprotein cholesterol tertile | | | *P*-value^†^ |
| --- | --- | --- | --- | --- |
|  | Lowest (n=81) | Intermediate (n=81) | Highest (n=80) |  |
| Baseline | 26 [6-35] | 34 [10-41] | 35 [21-65] | 0.001 |
| After 24 months | 29 [6-44] | 34 [11-51] | 41 [25-96] | 0.001 |
| *P*-value^§^ | 0.141 | 0.620 | 0.024 |  |
|  | Time-integrated triglyceride tertile | | | *P*-value^†^ |
|  | Lowest (n=81) | Intermediate (n=80) | Highest (n=81) |  |
| Baseline | 22 [6-36] | 26 [7-35] | 35 [20-66] | 0.003 |
| After 24 months | 24 [8-35] | 27 [7-37] | 37 [22-68] | 0.011 |
| *P*-value^§^ | 0.176 | 0.213 | 0.181 |  |
|  | Time-integrated high-density lipoprotein cholesterol tertile | | | *P*-value^†^ |
|  | Lowest (n=81) | Intermediate (n=81) | Highest (n=80) |  |
| Baseline | 31 [9-36] | 35 [8-58] | 35 [10-47] | 0.669 |
| After 24 months | 31 [6-35] | 37 [15-62] | 38 [11-69] | 0.458 |
| *P*-value^§^ | 0.713 | 0.134 | 0.061 |  |

Data are presented as median [interquartile range]. †*P*-values were obtained from linear regression analysis after adjustment for age, gender, and statin use. §*P*-values represent the radiographic score comparison between baseline and after 24 weeks. For comparison between the two groups, the Wilcoxon signed rank test was used.
