# Supplementary material for: LDL Cholesterolemia as a Novel Risk Factor for Radiographic Progression of Rheumatoid Arthritis: A Single-Center Prospective Study
Source: PLoS One. 2013 Jul 29;8(7):e68975. doi: 10.1371/journal.pone.0068975 (PMC3726747; doi:10.1371/journal.pone.0068975)
Supplement: Table S3 — Univariate analysis of clinical variables according to time-integrated LDL cholesterol levels. (DOCX) [file pone.0068975.s005.docx]

**Table S3**. Univariate analysis of clinical variables according to time-integrated LDL cholesterol levels

| Variables | Time-integrated LDL cholesterol tertiles | | | *P*-value |
| --- | --- | --- | --- | --- |
|  | 1^st^ (n=81) | 2^nd^ (n=81) | 3^rd^ (n=80) |  |
| Age, years | 52 [47-62] | 54 [43-61] | 53 [45-62] | 0.228 |
| Female, n (%) | 58 (71.6) | 64 (79.0) | 66 (82.5) | 0.204 |
| Body mass index, kg/m^2^ | 23.1 [20.4-25.4] | 22.5 [19.6-24.9] | 23.3 [20.7-24.7] | 0.211 |
| Disease duration, years | 6 [3-8] | 7 [4-15] | 7 [3-12] | 0.021 |
| Rheumatoid factor^§^, n (%) | 51 (63.0) | 57 (70.4) | 57 (71.3) | 0.051 |
| ACPA^§^, n (%) | 57 (70.4) | 63 (77.8) | 68 (85.0) | 0.048 |
| DAS28 | 3.7 [2.8-5.1] | 4.1 [3.1-5.6] | 4.3 [2.9-5.6] | 0.079 |
| Baseline ESR, mm/hour | 18 [11-34] | 26 [14-49] | 27 [15-44] | 0.120 |
| Time-integrated ESR | 792 [456-1752] | 816 [528-1296] | 840 [384-1824] | 0.039 |
| Baseline CRP, mg/dl | 0.18 [0.06-0.57] | 0.25 [0.12-1.16] | 0.39 [0.08-1.33] | 0.086 |
| Time-integrated CRP | 7.9 [2.4-34.8] | 10.3 [2.5-37.2] | 16.8 [4.7-49.3] | 0.028 |
| Glucocorticoid, n (%) | 62 (76.5) | 64 (79.0) | 61 (76.3) | 0.873 |
| Methotrexate, n (%) | 59 (72.9) | 61 (75.3) | 67 (83.8) | 0.032 |
| Hydroxychloroquine, n (%) | 53 (65.4) | 52 (64.2) | 50 (62.5) | 0.392 |
| Anti-TNFα, n (%) | 6 (7.4) | 13 (16.0) | 7 (8.8) | 0.209 |
| Statin, n (%) | 6 (7.4) | 13 (16.0) | 10 (12.5) | 0.211 |

Data are presented as median [interquartile range] or number (%). †P-values obtained from Kruskal-Wallis test. §=antibody positivity. LDL=low density lipoprotein, ACPA=anti-cyclic citrullinated peptide antibody, DAS28=disease activity score in 28 joints, ESR=erythrocyte sedimentation rate, CRP=C-reactive protein, and TNFα=tumor necrosis factor α. The positive cut-off value for ACPA was ≧5 U/ml.
