# Supplementary material for: LDL Cholesterolemia as a Novel Risk Factor for Radiographic Progression of Rheumatoid Arthritis: A Single-Center Prospective Study
Source: PLoS One. 2013 Jul 29;8(7):e68975. doi: 10.1371/journal.pone.0068975 (PMC3726747; doi:10.1371/journal.pone.0068975)
Supplement: Table S4 — Association between patient characteristics and radiographic severity at two years. (DOCX) [file pone.0068975.s006.docx]

**Table S4.** Association between patient characteristics and radiographic severity at two years

| **Variables** | **Univariate** | | **Multivariate** | |
| --- | --- | --- | --- | --- |
|  | γ | *P*-value | β | *P*-value |
| Age, years | 0.144 | 0.019 | 0.120 | 0.107 |
| Female, n (%) | 0.091 | 0.138 | NE | NE |
| Body mass index, kg/m^2^ | -0.178 | 0.004 | -0.127 | 0.095 |
| Disease duration, years | 0.497 | <0.001 | 0.444 | <0.001 |
| Rheumatoid factor^§^, n (%) | 0.103 | 0.334 | NE | NE |
| ACPA^§^, n (%) | 0.143 | 0.023 | 0.190 | 0.025 |
| DAS28 | 0.116 | 0.094 | NE | NE |
| Baseline ESR, mm/hour | 0.051 | 0.385 | NE | NE |
| Time-integrated ESR | 0.201 | 0.024 | 0.164 | 0.053 |
| Baseline CRP, mg/dl | 0.082 | 0.186 | NE | NE |
| Time-integrated CRP | 0.332 | <0.001 | 0.273 | 0.014 |
| Baseline SvdH score | 0.674 | 0.001 | 0.173 | 0.039 |
| Methotrexate, n (%) | 0.010 | 0.874 | NE | NE |
| Anti-TNFα, n (%) | -0.015 | 0.739 | NE | NE |
| Hydroxychlroloquine, n (%) | -0.021 | 0.732 | NE | NE |
| Statin, n (%) | 0.050 | 0.422 | NE | NE |
| Time-integrated LDL cholesterol | 0.235 | <0.001 | 0.154 | 0.046 |

SvdH=Sharp van der Heijde and NE=not entered. See the Supplementary Table 3 for other abbreviations. §Antibody positivity. The positive cut-off value for ACPA was ≧5 U/ml. γ=Spearman’s correlation coefficient. Multivariate model, adjusted R square=0.264, Model *P*<0.001
